# Supplementary figures and images for: Non-destructive lock-picking of a historical treasure chest by means of X-ray computed tomography
Source: PLoS One. 2020 Jul 6;15(7):e0235316. doi: 10.1371/journal.pone.0235316 (PMC7337312; doi:10.1371/journal.pone.0235316)

# Lock mechanism of a historical chest

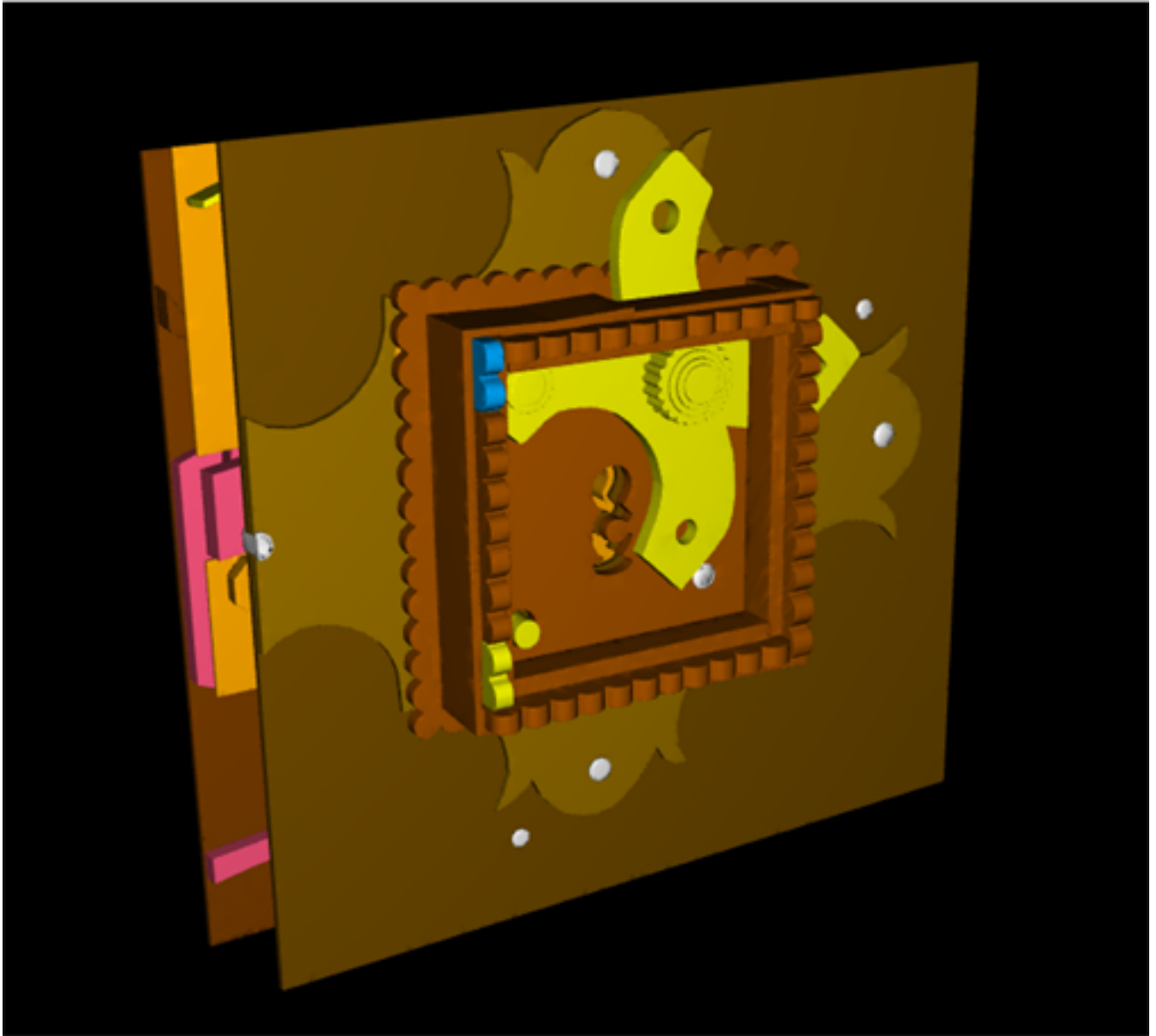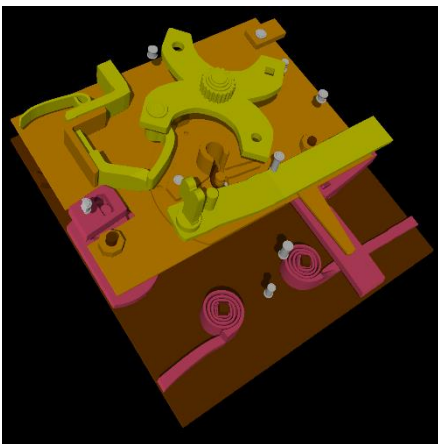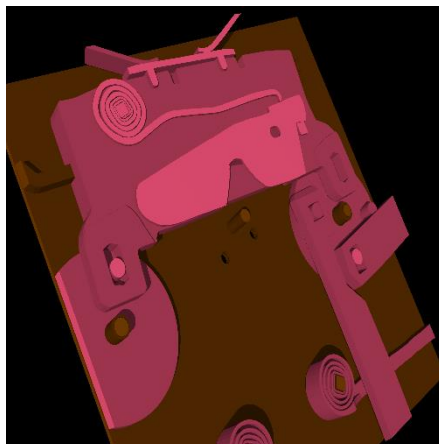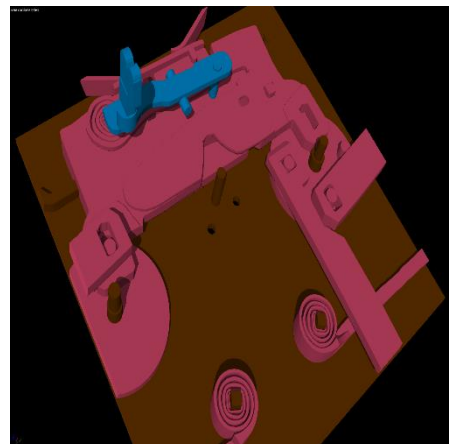

Default view

Supplement: S1 File — (PDF) [file pone.0235316.s001.pdf]
