# Supplementary material for: Non-destructive lock-picking of a historical treasure chest by means of X-ray computed tomography
Source: PLoS One. 2020 Jul 6;15(7):e0235316. doi: 10.1371/journal.pone.0235316 (PMC7337312; doi:10.1371/journal.pone.0235316)
Supplement: Attachment — Submitted filename: review_report.docx [file pone.0235316.s002.docx]

This paper reflects the results of an X-ray CT study for a very bulky object, a wooden box (chest) with a quite complicated locking device, which should be opened without damaging the structure after understanding the mechanism. The object has some cultural heritage value and the key is missing. In this respect, the work is an interesting collaboration of CT experts with persons from the restoration and museum side.

The paper is well written and structured and gives most of the details in order to understand the object’s structure and composition and the applied non-invasive technique, including the image processing steps.

The challenge of the study is the large size of the object (bigger than the field-of-view or beam diameter) and the thickness of the material layers to be penetrated. In this respect, the study is cutting-edge and the limitations and problems are highlighted accordingly.

Despite of the good quality of the submitted draft of the paper, there are some aspects to improve the manuscript further:

1. Abstract: this study is by far not “a new approach” (since CT is available since decades), but a nice demo for using the method at the limit of the beam penetration. Further, the application to cultural heritage objects and the interaction with museums people could be highlighted more.
2. More details should be given in the material and method description: which materials are involved in detail (Fe, Cu, wood, …); Why 1800 single projections were produced? What are the X-ray beam parameters (cone, focal spot, beam geometry, distances of source, sample, detector, …)? What is the performance of the reconstruction method?
3. Table 1: please, add also the thickness of the individual components of the lock!
4. Furthermore, it would be interesting to compare extracted virtual parts with the real parts of the lock – this describes the quality of the study.
5. Fig. 7 shows only the performance of the workshop … better show the digital layout derived from the CT volume data … and compare to reality.
6. Fig. 6 doesn’t give a global overview of the lock and its parts. A comparison of Fig. 4 with the “virtual reality” should be given. Better views and maybe dedicated slices should be added and explained. Otherwise, the study remains incomplete.
7. From the methodical point, it could be added, that the study was performed in a regime where the sample is larger than the beam as a FOV tomography run. Because of the high number of taken projections, the missing information by the highly attenuating range seems to be overcome?
