# Supplementary material for: Non-destructive lock-picking of a historical treasure chest by means of X-ray computed tomography
Source: PLoS One. 2020 Jul 6;15(7):e0235316. doi: 10.1371/journal.pone.0235316 (PMC7337312; doi:10.1371/journal.pone.0235316)
Supplement: Attachment — Submitted filename: Review_response.docx [file pone.0235316.s003.docx]

**Reviewer #1:** I like this interesting and curious paper and support its publication. The explanation of the work is clear, but it is unfortunately let down by poor written English throughout the paper. I feel that the paper needs to be thoroughly proof-read before it can be published.

>We had the paper proof-read by Martina Pořízková, née Horníčková. We attach her University Diploma in English.

It is not clear to me how figure 5 was generated. Presumably it was made with some 3D modelling software but was it made by hand after looking carefully at the CT scan or generated directly from CT?
> Figure 5 was generated directly from CT data, the method is briefly described in the lines 115 – 117 of the original manuscript. Nevertheless, we suggest a rephrased description: ”Individual parts of the lock mechanism were transformed into geometric objects which were subsequently colour-coded in a 3D visualization. The transformation into geometric objects was done manually. Based on the edges of the lock mechanism parts, polygonal formations were drawn using VG Studio software. ”

and add this also into the figure label, i.e. “3D model of the lock mechanism **from CT data** …”.

Please check PLOS's open data requirements. You say that the data are available from the author, but PLOS ONE might insist that you upload them to a repository.
>If PLOS ONE allows us to upload the data (5 – 10 GB) to the repository, we are happy to provide the original CT cross-sections. Since the 3D model is unfortunately bound to commercial Volume Graphics software, we introduce an interactive PDF which we include as a supplementary material.

**Reviewer #2:** This paper reflects the results of an X-ray CT study for a very bulky object, a wooden box (chest) with a quite complicated locking device, which should be opened without damaging the structure after understanding the mechanism. The object has some cultural heritage value and the key is missing. In this respect, the work is an interesting collaboration of CT experts with persons from the restoration and museum side.
The paper is well written and structured and gives most of the details in order to understand the object’s structure and composition and the applied non-invasive technique, including the image processing steps.
The challenge of the study is the large size of the object (bigger than the field-of-view or beam diameter) and the thickness of the material layers to be penetrated. In this respect, the study is cutting-edge and the limitations and problems are highlighted accordingly.
Despite of the good quality of the submitted draft of the paper, there are some aspects to improve the manuscript further:

1. Abstract: this study is by far not “a new approach” (since CT is available since decades), but a nice demo for using the method at the limit of the beam penetration. Further, the application to cultural heritage objects and the interaction with museums people could be highlighted more.

>The term “a new approach” in this case was used in connection to the historical chest opening, not in the sense of the CT technique utilization which, we agree, has been known for decades. As this is definitely not a common way of opening a locked object (no publication to our knowledge), we feel that it deserves to be marked as a novelty issue. However, the abstract was slightly modified as proposed.

2. More details should be given in the material and method description: which materials are involved in detail (Fe, Cu, wood, …); Why 1800 single projections were produced? What are the X-ray beam parameters (cone, focal spot, beam geometry, distances of source, sample, detector, …)? What is the performance of the reconstruction method?

>The whole chest is made of steel, i.e. Fe-C; which is written in line 87.

Regarding the CT measurement parameters, most of the requested details are not provided by the commercial CT systems, and the measurement according to some convenient standards would be out of the scope of this paper. Nevertheless, we upgraded the methodology and integrated parameters which are available.

- Number of projections is chosen based on the CT system recommendation reflecting the used detector width and rules for avoidance of a the undersampling effect.

- The focal spot size is not given for the micro-focus X-ray tube.

- The detector distance of 1255 mm and the object distance of 639 mm give a magnification of 1.9 and an angle of cone beam of 18°.

- The reconstruction method is generally based on a filtered back projection algorithm using the GE Datos|X 2.6 software but this is a commercial system and the specific method is not known.

3. Table 1: please, add also the thickness of the individual components of the lock!

>The thickness values were added to the Tab. 1.

Added thicknesses: case 3 mm, catcher 1.5 mm, arresting plate 6 mm, secret button 5 mm, X-shaped cap 5 mm, cradle 3.5 mm

4. Furthermore, it would be interesting to compare extracted virtual parts with the real parts of the lock – this describes the quality of the study.

>We don’t think it would bring anything new for the scope of the paper.

We have combined a presentation of 3D model data and photos for the most accurate and compact explanation of the mechanism functionality. The virtual data allow looking at its complexity via various clipping planes and the photos show the entire lock system including the parts which were not scanned.

In this study, we didn’t aim for an overall description of the lock mechanism, which is, however, a part of the restoration report (unfortunately not written in English in this case) which we properly reference.

5. Fig. 7 shows only the performance of the workshop … better show the digital layout derived from the CT volume data … and compare to reality.

>Unfortunately, the digital layout of the key was not created at all because the restorers basically used the shape of the key hole and the fundamental dimensions revealed by the CT data.

However, even if we had had the digital layout, it could only show whether the real key was or was not manufactured properly according to the virtual design.

6. Fig. 6 doesn’t give a global overview of the lock and its parts. A comparison of Fig. 4 with the “virtual reality” should be given. Better views and maybe dedicated slices should be added and explained. Otherwise, the study remains incomplete.

>This is actually our intention. Fig. 6 is focused only on an explanation of the position and movement of the components in contact with the key when it is turning. For this purpose, it is essential to watch the trajectory of each component and that is why the schematic drawing of the involved components is the best choice to make it as clear and straightforward as possible without any distractions. Furthermore, the information that the scheme concerns the first mechanism is given to the readers so they can easily connect it with the pink components in Fig. 5.

Moreover, providing a global overview is quite a difficult task, since only a small part is digitized by using CT. On the other hand, the photos of the complete lock system could have been taken only in the assembled stage.

Also, we are afraid, that the comparison of Fig. 4 with a virtual model would not bring any new information since we have scanned only the middle part around the key hole.

On the contrary, we realize that the position of the scanned area might be unclear, so we have marked it in Fig. 4. Furthermore, we introduce an interactive PDF document of the virtual model for a better illustration of the lock mechanism complexity in the 3D context. This will allow readers to rotate and move the lock. We would like to attach it as a supplementary material of this paper.

7. From the methodical point, it could be added, that the study was performed in a regime where the sample is larger than the beam as a FOV tomography run. Because of the high number of taken projections, the missing information by the highly attenuating range seems to be overcome?

>This regime is usually called a ROI tomography (Region Of Interest) by the CT community. https://doi.org/10.1016/j.compscitech.2019.107786

>In lines 110 – 111, it is already mentioned that we applied the CT scan only in the lock area (20 cm x 20 cm), however the ROI tomography reference was added.

>The high number of projections solves only the undersampling issue and reduces the noise.

Anyway, even a small amount of projections including total absorption (zero signal) will cause the broken edges of the parts.

**Reviewer #3:** The paper describes an interesting application of X-ray Computed Tomography to the non-destructive examination of the lock mechanism of a 19th century chest with missing key. The aim was to obtain a precise and detailed model of the lock mechanism in order to understand its functionality and to manufacture a new key copy for opening the chest without any damage. 3D-CT is nowadays a well-consolidated technique also in the field of Cultural Heritage and it has been applied in a lot of case-studies. However, the present study is quite new and interesting because of the nature of the analyzed object and the high radiopacity of its constituent material that typically may produce artifacts in the reconstructed images decreasing their quality. In this case the authors were able to obtain tomographic images of good quality, so to characterize completely the lock mechanism.
The paper is well written and the results are original, so I suggest to accept the manuscripts with minor revisions.

In the following, my observations and suggestions.

Introduction
Line 31: I suggest writing “The sample projections…..are….” instead of “The sample projection …is...” and to specify that the data are acquired over an angular range of 360 degrees.
Line 40: Unlike the Reference 17, the CT system used for the analysis mentioned in Reference 16 was developed by a research group in Bologna, not in Turin.
>The introduction was modified as proposed.

Materials and methods
Line 104: I suggest using “projections” instead of “positions”

>It was modified as proposed
Line 108: In my opinion, Reference 31 is not entirely relevant, as it does not report the use of metal filters to reduce scattering artefacts, as stated by the authors. Moreover, to my knowledge, metal filters are generally used to reduce beam hardening, while collimators are more useful to reduce scattered radiation.

>We agree with this knowledge and we have removed the scattering artefacts information from the text.
Lines 112-113: The sentence “Within this software…..was applied [32]” needs further explanations. In particular, what does it mean “…..in a different material mode (number 8.5)….”?

>The software uses two different settings – a single material mode and a different material mode. Based on our experience, the different material mode has better results when applied to dense materials. The number 8.5 is a value set in the software from the range of 1 to 10 and it is based on an earlier optimization (different values were tried out). The mode and the value are mentioned in order to provide as much information as possible to enable a repeating of the experiment.

Line 127: Fig. 4(a) should be replaced by Fig. 3(a)
>The section Materials and methods was modified as proposed.

References
Line 300: the word “editors” should be deleted
Line 328: Insert “;” between “organizace” and “2014”
Line 331: Insert “;” between “techniques” and “2012”.
>References were modified as proposed.

Finally, I suggest that the authors include (if possible) the data from the CT scan of the treasure chest as supplementary information.

>The data will be provided as a supplementary material.
